# Dietary Mannan Oligosaccharides Modulate Gut Microbiota, Increase Fecal Bile Acid Excretion, and Decrease Plasma Cholesterol and Atherosclerosis Development

**DOI:** 10.1002/mnfr.201700942

**Published:** 2018-05-17

**Authors:** Lisa R. Hoving, Saeed Katiraei, Marieke Heijink, Amanda Pronk, Lianne van der Wee‐Pals, Trea Streefland, Martin Giera, Ko Willems van Dijk, Vanessa van Harmelen

**Affiliations:** ^1^ Department of Human Genetics Leiden University Medical Center Leiden 2300 RC The Netherlands; ^2^ Einthoven Laboratory for Experimental Vascular Medicine Leiden University Medical Center Leiden 2300 RC The Netherlands; ^3^ Center for Proteomics and Metabolomics Leiden University Medical Center Leiden 2333 ZA The Netherlands; ^4^ Department of Medicine Division of Endocrinology Leiden University Medical Center Leiden 2333 ZA The Netherlands

**Keywords:** atherosclerosis, cholesterol, mannan oligosaccharides, microbiota, short‐chain fatty acids

## Abstract

**Scope:**

Mannan oligosaccharides (MOS) have proven effective at improving growth performance, while also reducing hyperlipidemia and inflammation. As atherosclerosis is accelerated both by hyperlipidemia and inflammation, we aim to determine the effect of dietary MOS on atherosclerosis development in hyperlipidemic ApoE*3‐Leiden.CETP (*E3L.CETP*) mice, a well‐established model for human‐like lipoprotein metabolism.

**Methods and results:**

Female *E3L.CETP* mice were fed a high‐cholesterol diet, with or without 1% MOS for 14 weeks. MOS substantially decreased atherosclerotic lesions up to 54%, as assessed in the valve area of the aortic root. In blood, IL‐1RA, monocyte subtypes, lipids, and bile acids (BAs) were not affected by MOS. Gut microbiota composition was determined using 16S rRNA gene sequencing and MOS increased the abundance of cecal *Bacteroides ovatus*. MOS did not affect fecal excretion of cholesterol, but increased fecal BAs as well as butyrate in cecum as determined by gas chromatography mass spectrometry.

**Conclusion:**

MOS decreased the onset of atherosclerosis development via lowering of plasma cholesterol levels. These effects were accompanied by increased cecal butyrate and fecal excretion of BAs, presumably mediated via interactions of MOS with the gut microbiota.

## 1 Introduction

Atherosclerosis is a major cause of severe disease in modern society and a leading cause of death.^1^ Left untreated, atherosclerosis leads to cardiovascular complications including heart attack and stroke. The development of atherosclerosis is initiated by LDL cholesterol deposition in the arterial wall, oxidation of these lipoproteins, and uptake by macrophages leading to foam cell formation.^2^ Accumulation of foam cells is associated with endothelial dysfunction, influx of inflammatory cells, and progression of atherosclerotic lesion formation. This process is further aggravated in the presence of systemic inflammation. Atherosclerosis is thus initiated by the formation of lesions within the arterial wall,^3^ and is driven by both lipids and by inflammation.^4^, ^5^, ^6^

Although relatively efficient drugs are available to inhibit the development of atherosclerosis, additional strategies that reduce inflammation and hyperlipidemia are urgently required. One potential candidate includes dietary supplementation with mannan oligosaccharides (MOS). MOS can be derived from the outer cell‐wall membrane of bacteria, plants, or yeast.^7^ Yeast *Saccharomyces cerevisiae*‐derived MOS have been widely used in livestock industry as an alternative to antibiotics and as food supplementation to ameliorate performance by reducing pathogenic contamination.^8^, ^9^, ^10^

Several studies demonstrated that MOS is able to inflammation, both within the gastrointestinal tract^11^ as well as systemically.^12^, ^13^ Additionally, in different studies using a variety of experimental animal models, it was shown that dietary supplementation with MOS lowered plasma cholesterol levels.^14^, ^15^, ^16^ However, the mechanism by which MOS exert their effect is not fully established. A suggested mode of action by which MOS may improve inflammation is via interaction and modification of the gut microbiota. According to Spring et al., MOS bind to type‐1 fimbriae of pathogenic bacteria and prevent their adherence to the intestinal mucosa,^17^ thereby reducing pathogen‐induced inflammation. Additionally, cholesterol levels might also be affected by the interaction of MOS with the gut microbiota. Gut microbiota play an important role in regulating bile acid (BA) metabolism by converting primary BAs to secondary BAs.^18^ Secondary BAs are relatively less efficiently reabsorbed and excreted more via the feces compared to primary BAs.^18^, ^19^, ^20^ Hepatic conversion of cholesterol to BAs balances fecal excretion, which is the major route for cholesterol catabolism and accounting for almost half of the cholesterol eliminated from the body per day.^21^ Therefore, differences in fecal BA excretion affects the enterohepatic circulation of cholesterol and may ultimately affect plasma cholesterol levels.^22^

Given the potential anti‐inflammatory and cholesterol‐lowering effects of MOS, we hypothesized that dietary MOS supplementation will reduce atherosclerosis development via interactions with the gut microbiota. In the present study, we set out to determine the effect of dietary MOS supplementation on systemic inflammation and plasma lipid levels in the progression of atherosclerosis, using female hyperlipidemic ApoE*3‐Leiden.CETP (*E3L.CETP*) mice, a well‐established mouse model for hyperlipidemia and atherosclerosis development.^23^, ^24^

We found that MOS modulated the gut microbiota composition and activity, which was associated with increased fecal BA excretion. Increased BA excretion can explain lowered plasma total cholesterol (TC) levels and subsequently decreased progression of atherosclerosis.

## 2 Experimental Section

### 2.1 Mice and Diet

Female *E3L.CETP* mice of 11–15 weeks of age were fed a control western‐type diet (WTD) containing 0.1% cholesterol (Diet T; AB Diets, Woerden, The Netherlands) or this diet supplemented with 1% MOS derived from *S. cerevisiae* (Actigen, Alltech, Ridderkerk, The Netherlands) for a total period of 14 weeks. After a run‐in period of 3 weeks with WTD, mice were randomized according to plasma total cholesterol, triglycerides (TG), body weight, and age. Mice were housed under temperature‐ and humidity‐controlled specific pathogen‐free (SPF) conditions with a 12:12 h light–dark cycle and free access to food and water. During the diet intervention, body weight and food intake were weekly measured. After 14 weeks of intervention, non‐fasted mice were sacrificed using CO_2_ inhalation, perfused with ice‐cold PBS through the heart, and trunk blood was collected via heart puncture. Livers were collected for further analysis. Mouse experiments were performed in compliance with Dutch government guidelines and the Directive 2010/63/EU of the European Parliament and had received approval from the University Ethical Review Board (Leiden University Medical Center, The Netherlands, permission no. 13164).

### 2.2 Atherosclerosis Quantification and (Immuno)Histochemical Analysis

After 14 weeks of dietary intervention, hearts were collected and fixed in phosphate‐buffered 4% formaldehyde, dehydrated in 70% ethanol, embedded in paraffin, and cross‐sectioned (5 μm) perpendicular to the axis of the aorta throughout the aortic root area, starting from the appearance of open aortic valve leaflets. Per mouse, four sections with 50 μm intervals were used for atherosclerosis quantification. Obtained sections were stained with hematoxylin phloxin saffron (HPS) for histological analysis. Lesions were visually categorized for lesion severity according to the guidelines of the American Heart Association adapted for mice.^25^ Various types of lesions were discerned: mild lesions (types 1–3), severe lesions (types 4 and 5), and the absence of lesions defined as “non‐diseased segments.” Rat monoclonal antimouse antibody MAC3 (1:1000; BD Pharmingen, SanDiego, CA, USA) was used to quantify macrophage area. Atherosclerotic lesion area and composition were analyzed using ImageJ software (NIH, Bethesda, Maryland, USA).

### 2.3 Flow Cytometry

Circulating monocytes were analyzed using flow cytometry. After lysis of red blood cells, pelleted cells were resuspended in FACS buffer and stained for 30 min at 4 °C in the dark with fluorescently labeled antibodies listed in Table 1, Supporting Information. Cells were measured on an LSR II flow cytometer using Diva 6 software (BD Biosciences, CA, USA). Data were analyzed using FlowJo software (Treestar, OR, USA). Representative gating schemes are shown in Figure S1, Supporting Information.

### 2.4 Plasma Parameters

At the indicated time points, 4 h‐fasted (from 8:00 a.m. to 12:00 p.m.) blood samples were collected by tail vein bleeding into chilled capillaries and isolated plasma was assayed for TC and TG using commercially available kits (Roche Diagnostics, Germany). For determination of plasma HDL‐cholesterol, apoB‐containing particles were precipitated from plasma with 20% polyethylene glycol in 200 mM glycine buffer (pH 10) and TC was measured in the supernatant. Non‐HDL was calculated by subtracting HDL values from TC values. Cholesterol exposure was calculated as the cumulative exposure over the number of weeks the WTD was fed. The plasma cytokine IL‐1RA was measured using the R&D Quantikine kit following the manufacturer's standard protocol (R&D Systems, Minneapolis, USA). Plasma concentrations of total BAs were determined using a colorimetric assay kit (Diazyme Laboratories, Poway, USA).

### 2.5 Liver Lipids

Lipids were extracted from the liver according to a protocol modified from Bligh and Dyer.^26^ Liver samples were homogenized in 10 μL ice‐cold CH_3_OH/mg tissue. Lipids were extracted by the addition of 1800 μL CH_3_OH:CHCl_3_ (3:1 v/v) to 45 μL homogenate and subsequent centrifugation. The homogenate was dried and dissolved in 2% Triton X‐100, and TC content was assayed as described above.

### 2.6 16S rRNA Gene Sequencing and Profiling

For 16S rRNA sequencing, genomic DNA was isolated from cecum samples and sent to the Broad Institute of MIT and Harvard (Cambridge, USA). Microbial 16S rRNA gene was amplified targeting the hyper‐variable V4 region using forward primer 515F (5’‐GTGCCAGCMGCCGCGGTAA‐3’) and the reverse primer 806R (5’‐GGACTACHVGGGTWTCTAAT‐3’). The cycling conditions consisted of an initial denaturation of 94 °C for 3 min, followed by 25 cycles of denaturation at 94 °C for 45 sec, annealing at 50 °C for 60 sec, extension at 72 °C for 5 min, and a final extension at 72 °C for 10 min. Sequencing was performed using the Illumina MiSeq platform generating paired‐end reads of 175 bp in length in each direction. Overlapping paired‐end reads were subsequently aligned. Details of this protocol are previously described.^27^

Raw sequence data quality was assessed using FastQC, version 0.11.2 (http://www.bioinformatics.babraham.ac.uk/projects/fastqc/). Reads’ quality was verified using Sickle version 1.33 (https://github.com/najoshi/sickle) and low‐quality reads were removed. For visualizing the taxonomic composition of the cecal microbiota and further β‐diversity analysis, QIIME, version 1.9.1 was used.^28^ In brief, closed reference OTU picking with 97% sequence similarity against GreenGenes 13.8 reference database was performed. Jackknifed β‐diversity of unweighted UniFrac distances, with 10 jackknifed replicates was measured at rarefaction depth of 5000 reads per sample.

### 2.7 Cecal Short‐Chain Fatty Acid Analysis

Cecum short‐chain fatty acid (SCFA) content was analyzed using GC‐MS as previously described with additional modifications.^29^ Briefly, aqueous extracts of cecal content were prepared and added to acetone along with the internal standards acetate‐d4, propionate‐d6, and butyrate‐d8. Subsequently, SCFAs were derivatized using pentafluorobenzyl bromide (PFBBr) (60 °C for 30 min). Samples were extracted by the addition of *n‐*hexane and water. The *n*‐hexane fraction was subjected for further analysis. A Bruker Scion 436 GC coupled to a Bruker Scion TQ MS (Bruker, Bremen, Germany) was employed. Injection was performed using a CTC PAL autosampler (CTC Analytics, Zwingen, Switzerland) splitless at 280 °C. The GC was equipped with an Agilent VF‐5ms (25 m × 0.25 mm i.d., 0.25 μm film thickness) column (Agilent, Waldbronn, Germany). The following temperature gradient was used: 1 min constant at 50 °C, linear increase at 40 °C/min to 60 °C, kept constant for 3 min, linear increase of 25 °C/min to 200 °C, linear increase at 40 °C/min to 315 °C, kept constant for 2 min. The transfer line and ionization source temperature were 280 °C. Methane 99.995% was used as chemical ionization gas and negatively charged molecular ions were detected in the selected ion monitoring mode.

### 2.8 Fecal Cholesterol and Bile Acid Analysis

Feces was collected over a 24‐h period for 3 consecutive days. Fecal samples were dried at room temperature, weighed, and homogenized. Fecal cholesterol, the fecal primary BAs cholic acid (CA), α‐muricholic acid (α‐MCA), and β‐muricholic acid (β‐MCA), and the secondary BAs hyocholic acid (HCA), deoxycholic acid (DCA), and ω‐muricholic acid (ω‐MCA) were determined by capillary gas chromatography on an Agilent gas chromatograph (HP 6890), equipped with a 25 m × 0.25 mm CP‐Sil‐19‐fused silica column (Varian, Middelburg, The Netherlands) and a flame ionization detector as described previously.^30^

### 2.9 RNA Isolation and qRT‐PCR

RNA was extracted from snap‐frozen liver samples using NucleoSpin RNA kit according to the manufacturer's instructions (Machery‐Nagel, Germany). Concentrations and purity of RNA were determined on a NanoDrop ND‐1000 spectrophotometer (Isogen, The Netherlands) and RNA was reverse‐transcribed using Moloney Murine Leukemia Virus Reverse Transcriptase (Promega, The Netherlands). The mRNA expression level of 7‐α‐hydroxylase (*Cyp7a1*) and sterol 27‐hydroxylase (*Cyp27a1*) were determined by qRT‐PCR, using SYBR green supermix (Biorad, The Netherlands) and the gene‐specific primers for *Cyp7a1* (forward: 5’‐CAGGGAGATGCTCTGTGTTCA‐3’; reverse: 5’‐AGGCATACATCCCTTCCGTGA‐3’) and for *Cyp27a1* (forward 5’‐TCTGGCTACCTGCACTTCCT‐3’; reverse: 5’‐CTGGATCTCTGGGCTCTTTG‐3’). mRNA expression was normalized to the housekeeping gene *36b4* (forward: 5’‐GGACCCGAGAAGACCTCCTT‐3’; reverse: 5’‐ GCACATCACTCAGAATTTCAATGG‐3’), and expressed as fold change versus control using the ΔΔ CT method.

### 2.10 Statistical Analysis

Data are presented as means ± SEM. Normal distribution of the data was tested using D'Agostino–Pearson omnibus normality test, and data were compared with the unpaired Student's *t*‐test in the case of normal distribution or with the nonparametric Mann–Whitney *U* test in the case of not normally distributed data. Correlation analysis was performed using linear regression analysis. The regression lines of the MOS‐supplemented mice versus control mice were compared to identify whether the correlations differed between the groups. First it was tested whether slopes of the lines differed and then whether intercepts of the lines differed. When the slopes and intercepts were not significantly different, linear regression analyses was performed on pooled data of both groups. *p *< 0.05 was considered as statistically significant. Analyses were performed using Graph Pad Prism version 7.0 (GraphPad Software, USA).

## 3 Results

### 3.1 MOS Decreased Atherosclerosis Development

**Figure 1:**
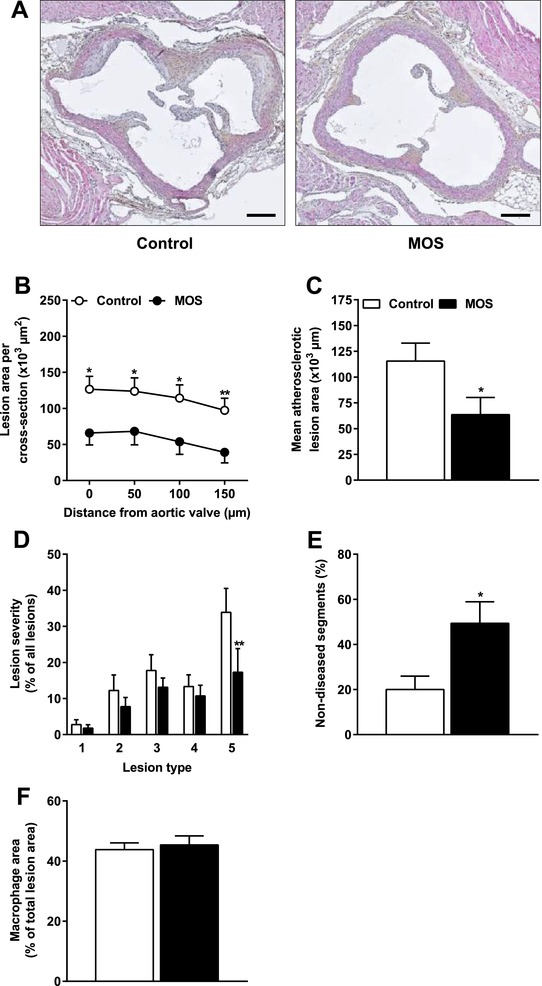
MOS decreased atherosclerosis development. Mice were fed a WTD with or without MOS for 14 weeks. A) Representative cross sections of the valve area of the aortic root stained with HPS are shown. Scale bar, 200 μm. B) Atherosclerotic lesion area was determined as a function of distance (50 μm intervals) starting from the appearance of open aortic valve leaflets covering 150 μm. C) The mean atherosclerotic lesion area was determined from the four consecutive cross sections, D) lesions were categorized according to lesion severity (type 1–5), E) the percentage of non‐diseased segments were scored, and F) macrophage area within the atherosclerotic lesions were quantified. Open bars/circles represent the control group and closed bars/circles represent the MOS group. Values are presented as means ± SEM (*n* = 14–15 mice per group). *p < 0.05, **p < 0.01 versus control.

To assess whether MOS affects atherosclerosis, we determined the progression of atherosclerosis in the aortic root after 14 weeks of MOS supplementation. As illustrated by representative images in **Figure** 1A, MOS markedly reduced the atherosclerotic lesion area throughout the whole aortic root (Figure 1B), which resulted in a 54% reduction of the mean atherosclerotic lesion area (*p *= 0.03; Figure 1C). Although the overall lesion severity was generally profound in both intervention groups, MOS greatly reduced type 5 lesions in the aortic root by 49% (*p *= 0.004; Figure 1D). Concomitantly, the number of non‐diseased segments was doubled after MOS supplementation (+147%; *p *= 0.01; Figure 1E). However, MOS did not affect the macrophage content of the atherosclerotic lesions of these mice (Figure 1F). Together, these findings demonstrated that MOS markedly delayed the progression of atherosclerosis and attenuated the severity of atherosclerotic lesions.

### 3.2 MOS did not Affect Markers of Systemic Inflammation

**Figure 2:**
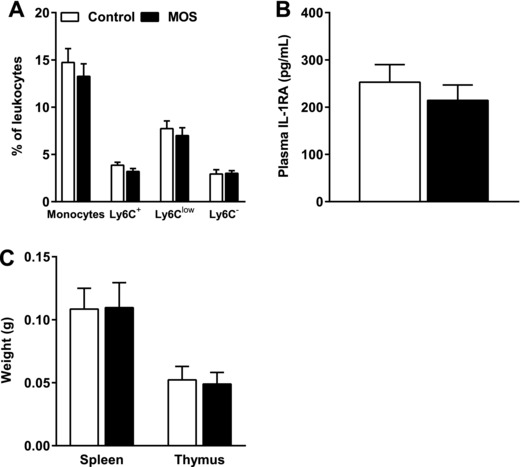
MOS did not affect markers of systemic inflammation. A) Circulating monocytes, Ly6C^+^, Ly6C^low^, and Ly6C^−^ monocyte subsets as a percentage of circulating leukocytes, B) plasma IL‐1RA levels, and C) spleen and thymus weight were measured in mice fed a WTD with or without MOS for 14 weeks. Open bars/circles represent the control group and closed bars/circles represent the MOS group. Values are presented as means ± SEM (*n* = 14–15 mice per group). *p *< 0.05 was considered as statistically significant.

We subsequently assessed whether the attenuation of atherosclerosis development and lesion severity after MOS supplementation was related to specific markers of systemic inflammation, such as the percentages of circulating monocytes (**Figure** 2A) and IL‐1RA (Figure 2B).^31^ Circulating monocytes and monocyte subsets (Ly6C^+^, Ly6C^low^, and Ly6C^−^) were not affected by MOS (Figure 2A). Finally, MOS did not alter spleen and thymus weight (Figure 2C). These results indicate that the MOS did not affect specific markers of systemic inflammation after 14 weeks of dietary intervention.

### 3.3 MOS Inhibited the Gradual Increase in Plasma Total Cholesterol Levels Without Affecting Plasma Triglycerides

**Figure 3:**
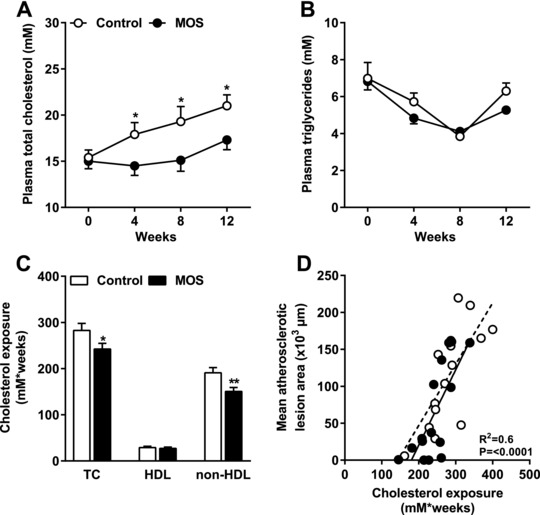
MOS inhibited the gradual increase in plasma total cholesterol levels without affecting plasma triglycerides. Mice were fed a WTD with or without MOS for 14 weeks. A) Plasma TC and B) TG were analyzed in 4‐h fasted mice at the indicated time points. C) Cumulative TC, HDL, and non‐HDL cholesterol exposure were calculated and TC exposure was plotted against mean atherosclerotic lesion area. D) The dotted line represents the regression line of the control mice and the straight line represents the regression line of the MOS‐supplemented mice. Open bars/circles represent the control group and closed bars/circles represent the MOS group. Values are presented as means ± SEM (*n* = 14–15 mice per group). *p < 0.05, **p < 0.01 versus control.

As MOS is known to beneficially affect hyperlipidemia, we determined the effect of MOS on plasma lipid levels. From week 4 onward, MOS significantly inhibited the gradual increase in plasma TC levels compared to the control group (**Figure** 3A) without affecting plasma TG levels (Figure 3B). In terms of cholesterol exposure, this reduction in TC after MOS supplementation was confined to a reduction in the non‐HDL cholesterol fraction (−21%; *p *= 0.008; Figure 3C). We performed regression analysis on TC exposure versus mean atherosclerotic lesion area. Comparison of the regression lines indicated that slopes (*F*_slopes_ = 0.34; *p* = NS) and intercepts (*F*_intercepts _= 0.77; *p* = NS) were similar for MOS‐supplemented mice and control mice (pooled data *R* ^2^ = 0.6; *p* < 0.0001; Figure 3D). This implies that the reduction in atherosclerotic lesion area after MOS supplementation was due to the cholesterol‐lowering effect of MOS.

### 3.4 The Cholesterol‐Lowering Effect of MOS Was not Due to Differences in Cholesterol Intake, Fecal Cholesterol Excretion, or Liver Cholesterol Levels

**Figure 4:**
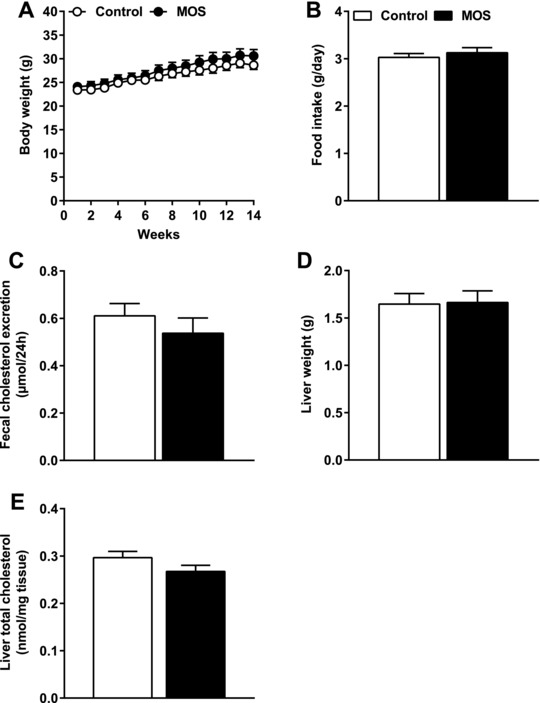
The cholesterol‐lowering effect of MOS was not due to differences in cholesterol intake, fecal cholesterol excretion, or liver cholesterol levels. A) Body weight, B) food intake, C) fecal cholesterol excretion, D) liver weight, and E) liver TC were determined in mice fed a WTD with or without MOS for 14 weeks. Open bars/circles represent the control group and closed bars/circles represent the MOS group. Values are presented as means ± SEM (*n* = 15 mice per group). *p *< 0.05 was considered as statistically significant.

Considering that plasma cholesterol levels might be affected by alterations in dietary cholesterol intake or fecal excretion, we assessed whether MOS affected these parameters. MOS did neither affect body weight (**Figure** 4A) nor food intake (Figure 4B), indicating that both groups ingested similar amounts of food and cholesterol via the diet. Furthermore, the fecal concentration of cholesterol was not different after MOS supplementation (Figure 4C), which demonstrates that fecal cholesterol excretion was comparable between the groups. Additionally, liver weight (Figure 4D) and liver TC (Figure 4E) were similar between the groups. Together, these data illustrate that the plasma cholesterol‐lowering effect of MOS was not due to reduced cholesterol intake, liver TC, or increased fecal cholesterol excretion.

### 3.5 MOS Increased the Abundance of Cecal *Bacteroides Ovatus* and Butyrate

**Figure 5:**
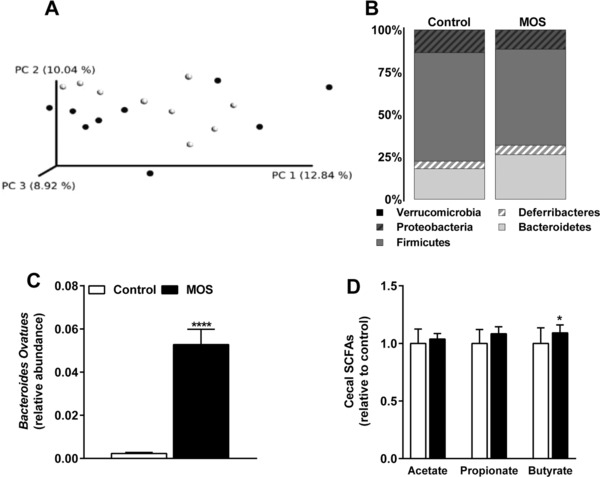
MOS increased the abundance of cecal *Bacteroides ovatus* and butyrate. A) Principal coordinates analysis plot of unweighted UniFrac distances of 16S rRNA gene sequences in which each circle represents an individual mouse. B) Microbiota composition at phylum level in cecal samples, C) relative abundance of *B. ovatus*, and D) the cecal SCFAs acetate, propionate, and butyrate of mice fed a WTD with or without MOS for 14 weeks. Open bars/circles represent the control group and closed bars/circles represent the MOS group. Values are presented as means ± SEM (*n* = 15 mice per group). *p < 0.05, **p < 0.01, ***p < 0.001, ****p < 0.0001 versus control.

MOS is thought to act in the gut via interactions with the gut microbiota. To decipher the effects of MOS on gut microbiota, we first determined the effect of MOS supplementation on gut microbiota composition and the relative abundance of specific microbial taxa by 16S rRNA gene sequencing. Clustering analysis of 16S rRNA gene sequences by unweighted UniFrac distances revealed no clustering based on intervention (**Figure** 5A), which indicated that the β‐diversity did not change after MOS supplementation. However, analysis of the gut microbiota at various taxonomic levels demonstrated that MOS altered the bacterial composition at phylum level, that is, MOS increased the abundance of *Bacteroidetes* (fold‐change = 1.5; *p* = 0.006; Figure 5B; **Table** 1) and decreased the abundance of *Firmicutes* (fold‐change = −1.1; *p* = 0.03)(Figure 5B; Table 1). At lower taxonomic levels, differences in microbial community between the control mice and the MOS‐supplemented mice became more apparent, although significant effects were mainly found on unidentified species (Table 1). One specific identified bacterium in the phylum of *Bacteroidetes* which significantly increased with 95% after MOS supplementation, was *B. ovatus* (fold‐change = 29.2; *p* = 0.0001; Figure 5C; Table 1). Therefore, t2he increase in the abundance of the phylum *Bacteroidetes* was mainly explained by an increase in *B. ovatus*. The decreased abundance of the phylum *Firmicutes* was mostly explained by a decrease in the order of *Clostridiales* (fold‐change = −1.2; *p* = 0.04; Table 1), the family *Lachnospiraceae* (fold‐change = −1.4; *p* = 0.03; Table 1), and unidentified taxonomic species in the genera of *Ruminococcus* (fold‐change = −2.3; *p* = 0.006; Table 1).

**Table 1:** Relative abundance of cecal microbiota

Phylum	Class	Order	Family	Genus	Species	Control [%]	MOS [%]	Fold change	*p*‐value^a^
Bacteroidetes						17.98	26.31	1.5	0.006
	Bacteroidia					17.98	26.31	1.5	0.006
		Bacteroidales				2.96	5.72	1.9	0.02
			Unidentified			1.63	5.50	3.4	0.0002
				*Unidentified*		1.83	5.50	3.0	0.0002
					*Unidentified*	1.83	5.50	3.0	0,0
			Bacteroidaceae			6.77	10.83	1.6	0.04
				*Bacteroides*		7.61	10.83	1.4	0.04
					*Unidentified*	7.38	5.55	−1.3	0.15
					*ovatus*	0.23	5.28	22.9	0.0001
			Porphyromonadaceae			0.84	1.54	1.8	0.19
				*Parabacteroides*		0.95	1.54	1.6	0.19
					*Unidentified*	0.95	1.54	1.6	0.19
			Rikenellaceae			2.26	5.18	2.3	0.01
				*Unidentified*		2.54	5.18	2.0	0.01
					*Unidentified*	2.54	5.18	2.0	0.01
			S24‐7			3.13	2.40	−1.3	0.16
				*Unidentified*		3.52	2.40	−1.5	0.16
					*Unidentified*	3.52	2.40	−1.5	0.16
			Paraprevotellaceae			1.36	0.86	−1.6	0.62
				*Prevotella*		1.53	0.86	−1.8	0.62
					*Unidentified*	1.53	0.86	−1.8	0.62
Deferribacteres						4.44	5.64	1.3	0.39
	Deferribacteres					4.44	5.64	1.3	0.39
		Deferribacterales				4.44	5.64	1.3	0.39
			Deferribacteraceae			3.94	5.64	1.4	0.39
				*Mucispirillum*		4.44	5.64	1.3	0.39
					*schaedleri*	4.44	5.64	1.3	0.39
Firmicutes						64.18	56.64	−1.1	0.03
	Bacilli					0.76	0.38	−2.0	0.16
		Lactobacillales				0.76	0.38	−2.0	0.16
			Lactobacillaceae			0.67	0.38	−1.8	0.16
				*Lactobacillus*		0.76	0.38	−2.0	0.16
					*Unidentified*	0.76	0.38	−2.0	0.16
	Clostridia					50.06	43.40	−1.2	0.04
		Clostridiales				50.06	43.40	−1.2	0.04
			Unidentified			31.05	31.46	1.0	0.12
				*Unidentified*		34.93	31.46	−1.1	0.12
					*Unidentified*	34.93	31.46	−1.1	0.12
			Lachnospiraceae			4.55	3.16	−1.4	0.03
				*Dorea*		1.23	0.67	−1.8	0.25
					*Unidentified*	1.23	0.67	−1.8	0.25
				*Ruminococcus*		3.89	2.49	−1.6	0.11
					*gnavus*	3.89	2.49	−1.6	0.11
			Peptostreptococcaceae			1.04	1.16	1.1	>.999
				*Unidentified*		1.16	1.16	1.0	>.999
					*Unidentified*	1.16	1.16	1.0	>.999
			Ruminococcaceae			7.85	7.62	1.0	0.35
				*Oscillospira*		7.20	6.92	1.0	0.81
					*Unidentified*	7.20	6.92	1.0	0.81
				*Ruminococcus*		1.64	0.70	−2.3	0.006
					*Unidentified*	1.64	0.70	−2.3	0.006
	Erysipelotrichi					13.36	12.86	1.0	0.90
		Erysipelotrichales				13.36	12.86	1.0	0.90
			Erysipelotrichaceae			11.88	12.86	1.1	0.90
				*Unidentified*		0.71	1.46	2.1	0.23
					*Unidentified*	0.71	1.46	2.1	0.23
				*Allobaculum*		12.66	11.40	−1.1	0.74
					*Unidentified*	12.66	11.40	−1.1	0.74
Proteobacteria						13.37	11.37	−1.2	0.47
	Deltaproteobacteria					13.37	11.37	−1.2	0.47
		Desulfovibrionales				13.37	11.37	−1.2	0.47
			Desulfovibrionaceae			11.89	11.37	1.0	0.47
				*Bilophila*		7.61	7.92	1.0	0.86
					*Unidentified*	7.61	7.92	1.0	0.86
				*Desulfovibrio*		5.76	3.45	−1.7	0.30
					*C21_c20*	5.76	3.45	−1.7	0.30
Verrucomicrobia						0.03	0.04	1.4	0.54
	Verrucomicrobiae					0.03	0.04	1.4	0.54
		Verrucomicrobiales				0.03	0.04	1.4	0.54
			Verrucomicrobiaceae			0.03	0.04	1.5	0.54
				*Akkermansia*		0.03	0.04	1.4	0.54
					*muciniphila*	0.03	0.04	1.4	0.54

*p *< 0.05 was considered statistically significant; MOS versus control

a Significance according to Mann–Whitney *U* test

We further assessed bacterial function by analyzing SCFAs in cecal content of these mice. MOS elevated cecal concentrations of the SCFA butyrate (+31%; *p* = 0.01; Figure 5C). Collectively, these data revealed that MOS altered the abundance of specific microbial taxa and modulated microbial function by increasing cecal butyrate.

### 3.6 MOS Increased Fecal Bile Acid Excretion

**Figure 6:**
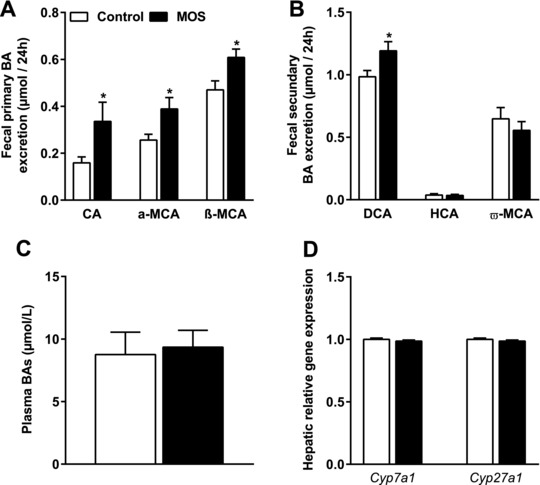
MOS increased fecal bile acid excretion. A) The fecal primary BAs cholic acid (CA), a‐muricholic acid (a‐MCA), β‐muricholic acid (β‐MCA), B) the fecal secondary hyocholic acid (HCA), deoxycholic acid (DCA), ω‐muricholic acid (ω‐MCA), C) plasma total BAs, and D) mRNA expression analysis of 7‐α‐hydroxylase (*Cyp7a1*) and sterol 27‐hydroxylase (*Cyp27a1*) were determined in mice fed a WTD with or without MOS for 14 weeks. Open bars/circles represent the control group and closed bars/circles represent the MOS group. Values are presented as means ± SEM (*n* = 8–15 mice per group). *p < 0.05 versus control.

Plasma cholesterol levels might be affected via changes in fecal BA excretion. Therefore, we determined whether MOS supplementation led to differences in fecal BA excretion. The concentration of the fecal primary BAs CA, α‐MCA, and β‐MCA were considerably increased after MOS supplementation (**Figure** 6A). For the secondary BAs, MOS increased the fecal excretion of DCA (Figure 6B). Despite increasing fecal BA excretion, MOS did not affect plasma BA concentrations (Figure 6C). We next performed mRNA analysis on *Cyp7a1* and *Cyp27a1*, the rate limiting enzymes in the major pathways for de novo BA synthesis. MOS did neither affect the expression of *Cyp7a1* nor *Cyp27a1* in the liver of these mice. Overall, we found that MOS increased the excretion of both primary and secondary BAs in feces without changing plasma BA levels and without affecting expression of *Cyp7a1* and *Cyp27a1*.

## 4 Discussion

Previous studies indicated that MOS decrease inflammation and plasma lipid levels.^12^, ^13^, ^14^, ^15^, ^16^ Here, we tested the hypothesis that MOS reduces atherosclerosis development via these pathways. We found that *S. cerevisiae*‐derived MOS indeed decreased the progression and severity of atherosclerosis in *E3L.CETP* mice. MOS reduced plasma cholesterol levels without affecting specific markers of systemic inflammation. Therefore, the decrease in atherosclerosis development after MOS supplementation can be explained by the cholesterol‐lowering effect of MOS.

MOS supplementation resulted in a reduction in plasma non‐HDL exposure by 21% and a decrease in total atherosclerotic lesion area in the aortic root by 54%. Similar reductions in plasma cholesterol and atherosclerotic lesion area have been achieved in *E3L.CETP* mice by statin treatment. For instance, low‐dose atorvastatin treatment led to a reduction in plasma cholesterol levels of 19% in *E3L.CETP* mice, accompanied by a reduction of ≈50% in the total atherosclerotic lesion area in the aortic root.^32^ In another study in *E3L.CETP* mice, rosuvastatin decreased plasma cholesterol by 25% and the total atherosclerotic lesion area by 62%.^33^ In this paper, rosuvastatin reduced atherosclerosis beyond and independent of the reduction achieved by cholesterol lowering alone, which may be at least partly explained by its anti‐inflammatory activity.^34^ Interestingly, the magnitude of the decrease in plasma cholesterol level and atherosclerotic lesion area by statins and MOS are similar indicating that MOS may also have pleiotropic effects beyond cholesterol lowering in the reduction of atherosclerosis.

We found that MOS specifically reduced the type 5 atherosclerotic lesions. In humans, these lesions are characterized as advanced and vulnerable lesions, which are susceptible to plaque rupture and to develop other cardiovascular complications such as coronary heart disease or ischemic stroke.^35^, ^36^ In addition, MOS‐supplemented mice displayed 29% more non‐diseased segments compared to the control group, indicating that MOS decreased de novo lesion formation in the aortic arch. Given the strong effects of MOS on both plasma TC and atherosclerosis development, comparable to the effects of statins in *E3L.CETP* mice, dietary MOS might present a novel approach in the prevention of atherosclerosis development and progression. It would be interesting to investigate whether MOS exerts its lipid‐lowering effect when supplemented on top of statins.

MOS did not affect specific markers of systemic inflammation associated with atherosclerosis,^31^ despite having anti‐inflammatory properties in previous studies.^11^, ^12^, ^13^ However, we cannot exclude the possibility that MOS might have modulated other systemic immune markers or that it has affected the immune status more subtly or locally, for example within the atherosclerotic plaque.

Spring et al. have proposed that MOS binds type‐1 fimbriae on pathogenic bacteria, preventing them from adhering to the intestinal mucosa and inducing an inflammatory trigger.^17^ In the majority of previous studies, anti‐inflammatory effects of MOS were observed after the application of pathogenic or pro‐inflammatory stimuli, such as *E. Coli*, *Salmonella*, or LPS.^11^, ^12^, ^13^ However, mice bred in our facility were kept under SPF conditions and therefore were not challenged with pathogenic stimuli. Whether a strong pathogenic stimulus is required to detect anti‐inflammatory effects of MOS, remains to be further investigated.

Previous studies have shown that dietary MOS alters the gut microbiota, although these studies were mainly conducted in other species such as chickens,^37^, ^38^ juvenile rainbow trout,^39^ or turkeys.^40^ In the present study conducted in mice, MOS also interacted with the gut microbiota as shown by an increase in butyrate levels in cecum as well as an increased abundance of the phylum *Bacteroidetes* and a decrease in the phylum *Firmicutes*. However, since the β‐diversity did not change, this suggests that MOS did not alter the microbial composition on a large scale. Notably, MOS induced the abundance of one specific identified species, *B. ovatus*. This bacterium is a well‐known mannan fermenter.^41^, ^42^, ^43^ Therefore, we suggest that MOS served as a substrate for *B. ovatus* to grow out. Interestingly, *B. ovatus* also expresses bile salt hydrolases (BSH)^44^, ^45^ and accordingly is able to deconjugate primary BAs into secondary BAs. Compared to primary BAs, secondary BAs are less efficiently reabsorbed in the intestine and are relatively more excreted via the feces.^18^, ^19^, ^20^ Indeed, MOS increased the fecal output of secondary BAs, likely via increasing the abundance of *B. ovatus*.

Regulation of plasma TC levels and BA metabolism are tightly linked as cholesterol from plasma serves as substrate for de novo BA synthesis in the liver.^46^, ^47^ Increased fecal excretion of BAs thus requires increased production of BAs. De novo synthesis of BA in mice and humans predominantly involves the enzymes *Cyp7a1* and *Cyp27a1*. Since we did not observe differences in the expression of *Cyp7a1* and *Cyp27a1*, it seems likely that the increased activity of both genes was due to post‐transcriptional regulation, alternative pathways involved in BA synthesis, or that other pathways in the enterohepatic circulation of BAs were affected.

In addition to increased secondary BA excretion, we also observed an increase in primary BA excretion after MOS supplementation. It is possible that MOS directly interacted with host cells involved in enterohepatic signaling, indirectly affecting recirculation of cholesterol or BAs, or that MOS acted as a BA sequestrant leading to reduced reabsorption of BAs. However, this seems unlikely since BA sequestrants usually result in increased plasma TG levels,^48^ which we did not observe in our study.

In literature, there are indications that the SCFA butyrate is associated with plasma cholesterol levels. In a previous study performed in mice, cecal infusion of butyrate increased hepatic cholesterol synthesis, suggesting that butyrate might elevate plasma cholesterol.^49^ However, in another study performed in rats, hepatic cholesterol synthesis was reduced after ingestion of butyrate, albeit butyrate was given in combination with other SCFAs.^50^ It remains to be determined whether increased cecal butyrate levels are involved in the cholesterol‐lowering effect of MOS.

In conclusion, MOS decreased the progression of atherosclerosis up to 54% in *E3L.CETP* mice, which was largely explained by a reduction in plasma non‐HDL cholesterol. The cholesterol‐lowering effect of MOS was accompanied and likely explained by modulation of the gut microbiota, increased cecal butyrate levels, and increased fecal BA excretion.

## Conflict of Interest

The authors declare no conflict of interest.

## Supporting information

Supporting Information
