# Supplementary material for: Dietary Mannan Oligosaccharides Modulate Gut Microbiota, Increase Fecal Bile Acid Excretion, and Decrease Plasma Cholesterol and Atherosclerosis Development
Source: Mol Nutr Food Res. 2018 May 17;62(10):1700942. doi: 10.1002/mnfr.201700942 (PMC6001637; doi:10.1002/mnfr.201700942)
Supplement: Supplementary file 1 — Supporting Information [file MNFR-62-na-s001.docx]

SUPPORTING INFORMATION FOR ONLINE PUBLICATION

**Dietary mannan oligosaccharides modulate gut microbiota, increase fecal bile acid excretion, and decrease plasma cholesterol and atherosclerosis development**

Lisa R. Hoving et al.

## Table 1. Antibodies used for flow cytometry

| **Antibody** | **Fluorochrome** | **Dilution** | **Clone, supplier** |
| --- | --- | --- | --- |
| CD45.2 | FITC | 1:100 | 104, BioLegend |
| CD11b | Pacific Blue | 1:150 | M1/70, BioLegend |
| CD115-Biotin  Streptavidin | n.a.  PeCy5 | 1:100  1:100 | AFS98, eBioScience  SAV, eBioScience |
| GR-1 | PeCy7 | 1:1500 | RB6-8C5 |

## Figure 1. Gating strategy


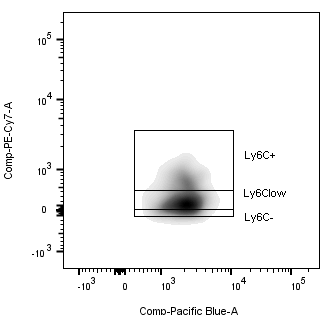

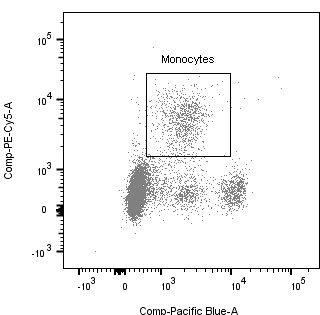


CD11b

GR-1

CD11b

CD115

**A**

**B**

Gating strategies for the analysis of total monocytes [A], Ly6C^+^, Ly6C^low^, and Ly6C^-^ monocyte subsets [B] in whole blood.
